# Safety and efficacy of transcatheter embolization for pulmonary arteriovenous fistula: a 21-year retrospective study

**DOI:** 10.3389/fcvm.2026.1732994

**Published:** 2026-05-29

**Authors:** Hong Lu, Jianming Wang, Jiawang Xiao, Zhongchao Wang, Hui Yao, Benshen Li, Yuan Mi, Qiguang Wang

**Affiliations:** Department of Congenital Heart Disease, General Hospital of Northern Theater Command, Shenyang, China

**Keywords:** embolization, interventional therapy, pulmonary arteriovenous fistula, retrospective study, transcatheter embolization

## Abstract

**Background:**

This study aimed to evaluate the safety and efficacy of transcatheter embolization (TCE) for pulmonary arteriovenous fistula (PAVF).

**Methods:**

From January 1, 2003 to December 31, 2024, 28 patients (13 male, 15 female) with 41 PAVFs underwent TCE. Changes in arterial oxygen saturation (SaO_2_), procedural success rate, reperfusion rate, and complications were evaluated.

**Results:**

The procedural success rate was 100%, with an immediate complete occlusion rate of 87.8%.Postoperative SaO2 was significantly higher than that before operation (92.0% vs. 84.1%, *P* < 0.001). The 41 PAVFs were divided into four groups (coil group [*n* = 6], MemoPart PDA occluder group [*n* = 22], ADO II group [*n* = 7], and AVP I group [*n* = 6]). The average diameter of the arteries was 3.17 ± 0.7 mm, 11.86 ± 3.31 mm, 5.98 ± 2.54 mm, and 8.72 ± 3.78 mm, respectively, showing a statistically significant difference between groups (*P* < 0.001). The average clinical follow-up time was 19.46 ± 26.04 months (range: 6–162 months). During the follow-up period, 2 patients (7.14%) developed fever and 1 patient (3.57%) developed pleural effusion. 1 case (14.29%) of PAVFs experienced reperfusion in the ADO II group, while reperfusion did not occur in the other three groups, and the difference was not statistically significant (*P* = 0.463).

**Conclusion:**

The adaptable utilization of various occluders can enhance the success rate of achieving effective occlusion. TCE for PAVFs demonstrates high safety and efficacy, with excellent procedural success rates and sustained long-term outcomes.

## 1 Introduction

Pulmonary arteriovenous fistulas (PAVFs) are rare pulmonary vascular malformations characterized by abnormal direct connections between pulmonary arteries and veins, which circumvent the capillary network. This abnormality results in a low-resistance, high-flow right-to-left shunt within the pulmonary circulation. The incidence of PAVFs is estimated to be 2–3 per 100,000 individuals, with a male-to-female ratio ranging from approximately 1:1.5–1:1.8 (1). Pathologically, PAVFs are categorized into simple, complex, and diffuse types. Simple-type PAVFs consist of a single feeding artery that connects directly to a single draining vein via an aneurysmal sac, without the presence of intervening capillaries. In contrast, complex-type PAVFs involve two or more feeding arteries and/or two or more draining veins interconnected through a septated aneurysmal sac or vascular network. The diffuse-type is characterized by extensive capillary telangiectasias lacking discrete aneurysmal sacs, resulting in numerous microscopic arteriovenous communications that are diffusely distributed throughout the lung parenchyma. These lesions may be solitary or multiple, with the majority of isolated PAVFs occurring in the lower lobes, particularly in the left lower lobe, which is the most frequently affected region (2). Clinical manifestations are largely contingent upon the size and number of lesions, as well as the resultant shunts. Patients may be asymptomatic, or may present with symptoms such as hypoxemia, hemoptysis, acute ischemic stroke, brain abscess and migraine (3). The incidence of PAVFs is associated with hereditary hemorrhagic telangiectasia (HHT), although it may also arise secondary to acquired conditions such as liver cirrhosis/hepatopulmonary syndrome, chest trauma, and infections (4). PAVFs frequently coexist with congenital heart diseases and cardiovascular malformations, including atrial septal defect, patent foramen ovale, and partial anomalous pulmonary venous return, which further exacerbate the risk of shunting and embolism. Clinically, it is imperative to differentiate PAVFs from pulmonary venous malformation, pulmonary hypertension, pulmonary embolism, pneumonia, lung tumors, pulmonary sequestration, and cyanotic congenital heart disease. A definitive diagnosis can be established through a combination of imaging modalities (CTA/MRA/angiography), echocardiography, and clinical history (5, 6).

The standard therapeutic intervention for PAVFs is transcatheter embolization(TCE), which aims to achieve durable and complete occlusion (7). In comparison to surgical treatment, interventional therapy presents several advantages including minimized trauma, expedited recovery, a definitive therapeutic effect, and reduced rates of complications. Current studies on PAVFs predominantly comprises case reports and small-series analyses. Critical unresolved issues include the criteria for device selection based on the size, multiplicity, and anatomical location of fistulae; the long-term outcomes associated with various embolization strategies; and the stratification of reperfusion risk according to hemodynamic factors. Consequently, this study aims to evaluate the long-term safety and efficacy of transcatheter embolization for PAVFs, and to investigate the application strategies of different embolic materials in PAVFs with diverse morphologies.

## 2 Materials and methods

### 2.1 Patients

A total of 28 patients with PAVFs who underwent interventional embolization at the Department of Congenital Heart Disease, General Hospital of Northern Theater Command from January 1, 2003 to December 31, 2024 were enrolled in the study. The diagnosis of pulmonary arteriovenous fistula was established using chest computed tomography angiography (CTA) and digital subtraction angiography (DSA). The inclusion criteria encompassed patients presenting with any surgical indication for PAVFs including single cystic PAVFs. For multiple cystic PAVFs, the approach involved sequential embolization targeting feeding arteries with larger shunt volumes. In cases of diffuse PAVFs, the embolization could be directed towards the more severely affected pulmonary lobe, with the possibility of multiple procedures. Patients were included if surgical treatment was deemed difficult, high-risk, or contraindicated, or if there were recurrences or residual lesions post-surgery. Additionally, patients were required to have no other conditions necessitating surgical intervention and to be free from infections, thromboembolism, or other contraindications to interventional therapy within the preceding month. Exclusion criteria comprised included: patients with severe cardiopulmonary insufficiency that precluded tolerance of the intervention, those with moderate or severe pulmonary hypertension, particularly if a significant increase in pressure was observed following balloon catheter trial occlusion of the feeding artery, and individuals with incomplete follow-up data or an inability to comply with follow-up requirements. The study received approval from the Ethics Committee of the General Hospital of Northern Theater Command [Ethics Approval Number: Lun Shen Y (2025) 082], and informed consent was obtained from each patient before TCE.

### 2.2 Percutaneous embolization procedure

Prior to the initiation of treatment, all patients underwent a thorough clinical assessment. Baseline imaging data included at least one of the following modalities, as CTA, contrast-enhanced CT scan, non-contrast CT scan, or chest x-ray. Standard laboratory evaluations were conducted, encompassing complete blood count, coagulation screening, and liver function tests, to exclude the presence of hepatopulmonary syndrome. Prophylactic antibiotics were administered preoperatively, and comprehensive preoperative preparation was ensured. Under local anesthesia with 1% lidocaine, a puncture of the right femoral vein was performed. Following systemic administration of heparin, a guidewire was introduced sequentially inserted, facilitating the placement of inner and outer sheaths. A 5F or 6F pigtail catheter was advanced through the right femoral vein into right atrium, right ventricle, and subsequently into the left and right pulmonary arteries. Measurements of pulmonary artery pressure (PAP) and right atrial pressure (RAP) were obtained. Anteroposterior and lateral angiography of the bilateral pulmonary arteries was performed to assess the morphology, size, and trajectory of the fistula, thereby identifying target vessels for embolization and precisely measuring vascular diameters. The selection of different types of devices is contingent upon vascular anatomy, vessel size, and operator's preference. To achieve optimal occlusion, it is imperative to select an appropriate device according to the oversize principle. Five minutes after the device implantation, repeat angiography was performed. Surgical success is defined as the effective delivery and implantation of various closure devices at the target site of the pulmonary arteriovenous fistula via an interventional approach. Immediate complete closure refers to the absence of residual shunting confirmed by angiography, indicating complete occlusion. Subsequently, the embolic device was released. Arterial oxygen saturation (SaO_2_) via femoral artery was measured pre- and post-procedure. Upon completion, catheters were withdrawn, and local compression bandaging was applied.

**Figure 1:**
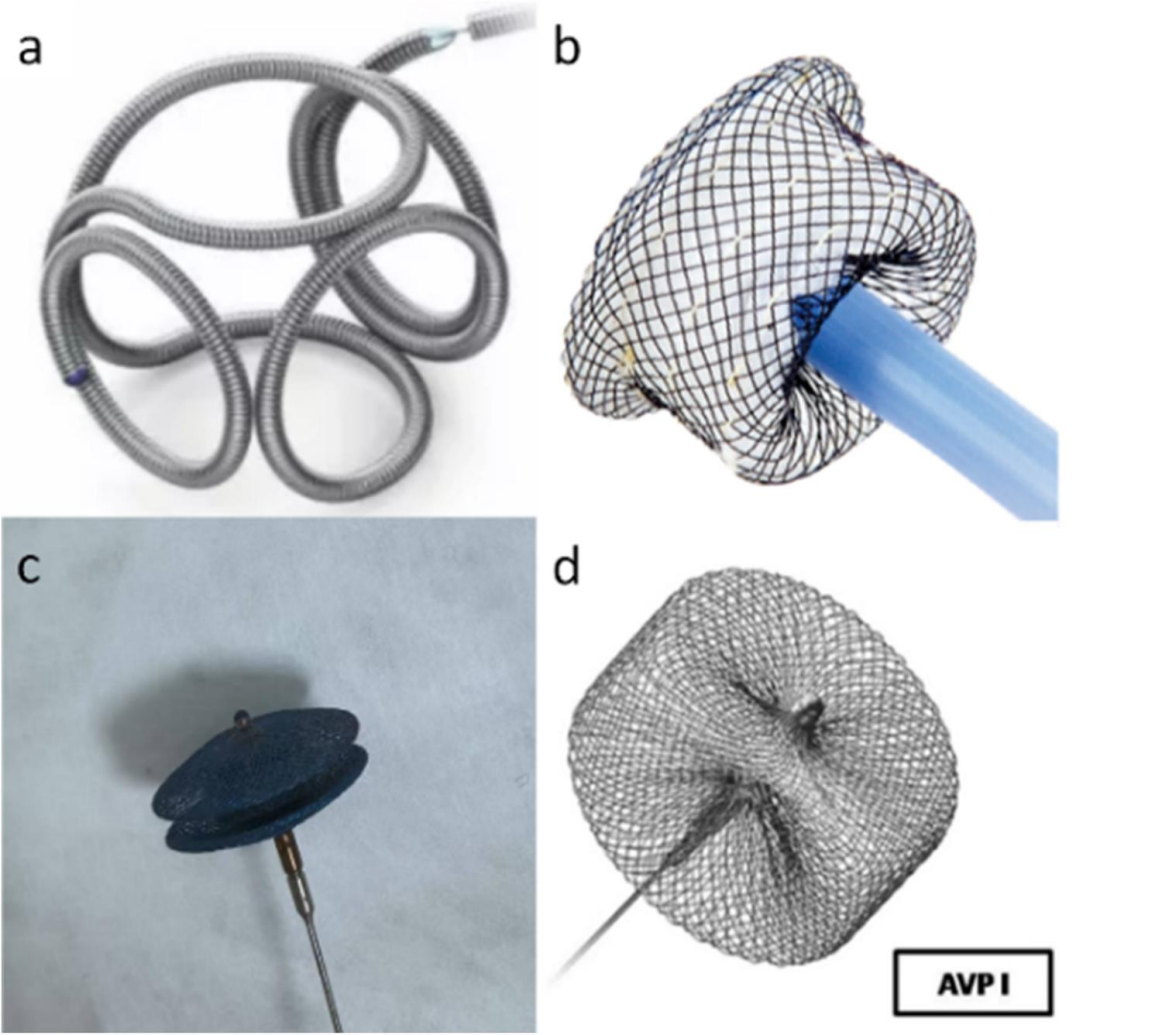
**(a)** Axium™ bare coil (ev3, Inc., USA); **(b)** MemoPart PDA Occluder (Shanghai Shape Memory Alloy Co., Ltd., China); **(c)** ADO Ⅱ (AGA Medical, USA); **(d)** AVP Ⅰ (AGA medical, USA).

The embolic materials utilized in this study included coils, MemoPart PDA occluders, the Amplatzer Duct Occluder type II (ADO II), and the Amplatzer Vascular Plug type Ⅰ (AVP Ⅰ) (Figure 1). All these embolic materials are mechanically detachable, allowing for precise positioning and release. The coil (ev3, Inc., USA) is an Axium™ Bare coil, which consists of a platinum-tungsten alloy wire core, polypropylene fiber strands, and a 316L stainless-steel detachment zone, with 3D diameters ranging from 2 to 25 mm. This model is a widely used detachable coil in China (8). The MemoPart PDA Occluder (Shanghai Shape Memory Alloy Co., Ltd., China) is fabricated from nitinol woven into a three-dimensional structure, featuring three layers of polyester fabric within the stent, and features a double-riveted self-expanding design. Its aortic disc diameter ranges from 6 to 22 mm. The ADO Ⅱ (AGA Medical, USA) is constructed with nitinol woven into a dumbbell-shaped configuration, connected by a central waist region without polyester filling. It provides six potential occlusion planes and is terminated with screw joints, with a waist diameter of 3–6 mm and a disc diameter of 9–12 mm. The AVP Ⅰ (AGA Medical, USA) is formed by a single-layer nitinol wire mesh, deployed into a cylindrical net structure without membrane coverage and its size is 4–16 mm.

### 2.3 Follow-up protocol section

Postoperative monitoring includes chest x-ray and echocardiography at 1, 3, and 6 months. CTA is recommended annually, beginning 6 months after the procedure (9, 10). The symptom relief was recorded in detail at each follow-up. Detailed documentation of symptom relief is conducted at each follow-up. In cases where reperfusion or enlargement of PAVFs is suspected, pulmonary angiography is advised, with embolotherapy administered if necessary. Specialists monitor changes in patients' symptoms through outpatient follow-ups and telephone consultations.

Serious complications were defined as death, air embolism, non-targeted embolization, pulmonary infarction, pulmonary hypertensive crisis, cardiac perforation, and severe arrhythmia. Minor complications include pleuritic chest pain, transient bradycardia, minor hemoptysis, minor atelectasis, minor pneumothorax, etc.

### 2.4 Statistical analysis

Data were analyzed using SPSS 27.0 software. Quantitative data conforming to the normal distribution were expressed as mean ± standard deviation $\left(\bar{x}\pm s\right)$ while non-normally distributed measures were expressed as median and quartile [M(P25, P75)]. For normally distributed continuous data with homogeneity of variance, intergroup comparisons utilized paired *t*-tests or one-way analysis of variance (ANOVA); otherwise, Wilcoxon's rank-sum test or Kruskal–Wallis test was used. Qualitative data were expressed in terms of frequency and percentage (%), and comparisons between groups were made using the chi-square test, the corrected chi-square test, or Fisher's exact test. Take *P* < 0. 05 as a statistical significance.

## 3 Results

### 3.1 Baseline characteristics

The clinical characteristics of the patient are summarized in Table 1. The study cohort comprised 28 patients (13 males, 15 females) with a mean age of 27.2 ± 17.6 years (range: 2–60 years). Among the 28 patients, 4 (14.29%) had mild tricuspid regurgitation, and 1 (3.57%) had a carotid cavernous fistula (CCF). No patients were diagnosed with systemic arterial hypertension (HTN), diabetes mellitus, coronary artery disease (CAD), chronic obstructive pulmonary disease (COPD), or congenital heart defects (CHD). Echocardiographic evaluation revealed a mean left ventricular ejection fraction (LVEF) of 66.32 ± 4.90% and a mean left atrial volume (LAV) of 28.12 ± 4.85 mL. 14 patients (50%) had single PAVFs and 14 patients (50%) had multiple PAVFs. Pulmonary angiography revealed 69 PAVFs. Most were located in the lower lobes: 16 (23.19%) in the right lower lobe, 9 (13%) in the right middle lobe, and 7 (10.14%) in the right upper lobe, 19 (27.54%) the left lower lobe, and 18 (26.09%) in the left upper lobe. 35 (50.72%) PAVFs were simple type, 11 (15.94%) were complex type, and 23 cases (33.33%) were diffuse type.

**Table 1:** Descriptive statistics on patients and PAVFs .

Variables	Patients (*N* = 28)
Age (years): mean ± SD (Range)	27.2 ± 17.6 (2–60)
Sex (male: female ratio), *n*	13/15
Presentation: *n*/*N* (%)	
Dyspnea	13/28 (46.43)
Hemoptysis	3/28 (10.71)
Cyanosis	13/28 (46.43)
Migraine	7/28 (25.00)
Stroke	5/28 (17.86)
Comorbidities	
Tricuspid regurgitation	4/28 (14.29)
Other vascular malformation	1/28 (3.57)
Echocardiographic parameters	
LVEF (%)	66.32 ± 4.90
LAV (mL)	28.12 ± 4.85
SaO_2_(%): mean ± SD (Range)	83.72 ± 10.15 (66–97.2)
Treated PAVFs number:	41/69
Follow-up period (months): mean ± SD (Range)	19.46 ± 26.04 (6–162)

PAVFs, pulmonary arteriovenous fistulas; SaO_2_, arterial oxygen saturation; LVEF, left ventricular ejection fraction; LAV, left atrial volume.

### 3.2 PAVFs occlusion

**Figure 2:**
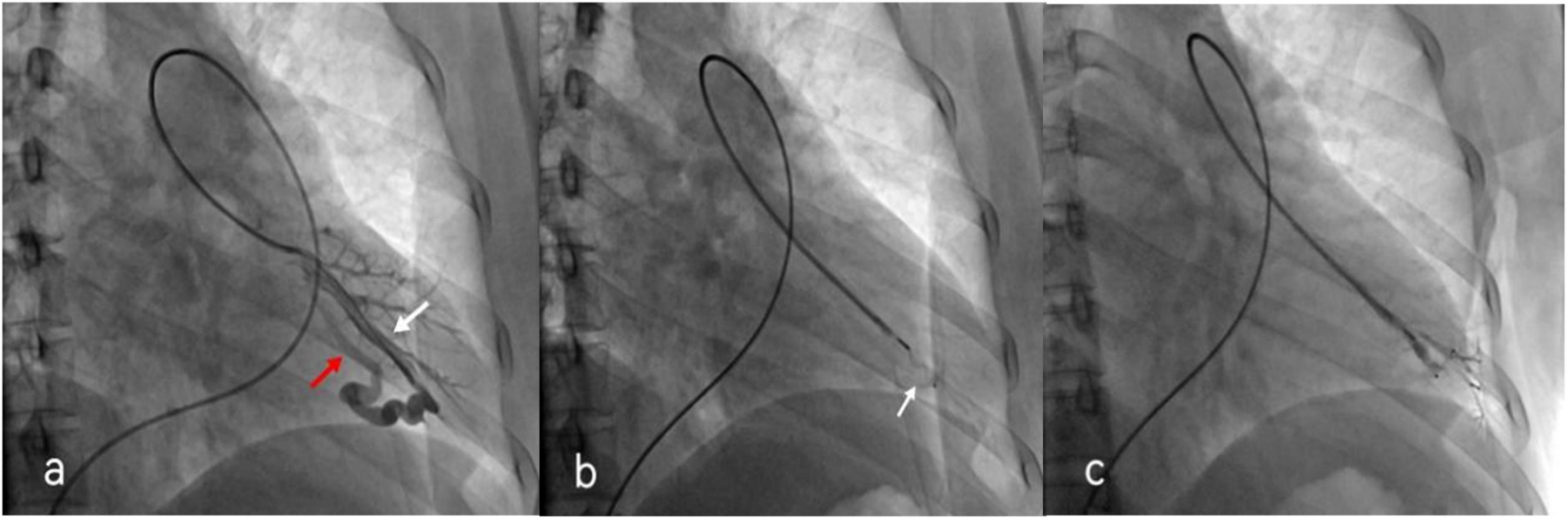
**(a)** Selective pulmonary angiography demonstrates a dilated and tortuous artery (white arrow) connected to a draining vein (red arrow). **(b)** Deployment of an ADO Ⅱ occluder (Model: 9-PDA2-03-04, AGA Medical) at the fistula site (arrow). **(c)** After 5 min, repeat contrast injection shows no significant contrast filling distal to the occluder, confirming complete occlusion.

41 PAVFs were embolized: 6 PAVFs (14.63%) with coils, 22 (53.66%) with MemoPart PDA occluders, 7 (17.07%) with the ADO II (Figure 2), and 6 (14.63%) with the AVP I. The mean pulmonary artery pressure (mPAP) was 17.71 ± 4.12 mmHg (range: 12–26.67 mmHg), and the mean right atrial pressure (mRAP) was 4.68 ± 2.22 mmHg (range: 2.33–12 mmHg). There were no significant intergroup differences (all *P* > 0.05), see Supplementary Table S1 for details. The mean diameter of the feeding arteries was 9.12 ± 4.44 mm (range: 2.48–16.4 mm). Procedure time was 82.27 ± 27.96 min (range: 21–135 min), with irradiation duration of 24.04 ± 10.77 min (range: 4.51–47.55 min). The technical success rate was 100%, and the immediate complete occlusion rate was 87.8%. 41 PAVFs were divided into four groups [coil group (*n* = 6), MemoPart PDA occluder group (*n* = 22), ADO II group (*n* = 7), and AVP I group (*n* = 6)]. There were statistically significant differences in the mean diameters of blood vessels between the four groups (*P* < 0.001), which were 3.17 ± 0.7 mm (range: 2.48–4.09 mm), 11.86 ± 3.31 mm (range: 6.42–16.4 mm), 5.98 ± 2.54 mm (range: 3.64–10.5 mm), 8.72 ± 3.78 mm (range: 4.2–12.7 mm), respectively. The ratio of device diameter to target vessel diameter for the coil group, MemoPart PDA occluder group, ADO II group, and AVP I group was 1.51 ± 0.34, 1.40 ± 0.26, 1.95 ± 0.79, and 1.36 ± 0.18, respectively. The procedure and irradiation time varied substantially between the four groups (Table 2). The pathological classification of PAVFs in the coil group and AVP I group was simple (100%); MemoPart PDA occluder group was simple in 8 cases (36.36%), complex in 5 cases (22.72%), diffuse in 9 cases (40.9%); and 4 (57.14%) simple type in the ADO II group, 2 (28.57%) complex type, and 1 (14.29%) diffuse type. The immediate complete occlusion rate was 100% in the coil group and the ADO II group. The MemoPart PDA occluder group and the AVP I group had 3 cases (13.64%) and 2 cases (33.33%), respectively, where a small amount of contrast agent remained after PAVF occlusion.

**Table 2:** The diameter of feeding arteries, procedure time and irradiation duration of PAVFs in four groups.

Parameters	Coil	MemoPart PDA occluder	ADO Ⅱ	AVP Ⅰ	H-value	*P*-value
Diameter of feeding artery (mm)	3.17 ± 0.70	11.86 ± 3.31	5.98 ± 2.54	8.72 ± 3.78	23.53	<0.001
Embolization device size	4.67 ± 0.82	16.91 ± 3.99	10.71 ± 1.38	11.67 ± 4.63	25.13	<0.001
Device-to-vessel diameter ratio	1.51 ± 0.34	1.40 ± 0.26	1.95 ± 0.79	1.36 ± 0.18	5.93	0.115
Procedure time (min)	95.83 ± 15.30	71.05 ± 30.13	80.57 ± 12.81	108.33 ± 20.41	11.10	0.011
Irradiation duration (min)	37.14 ± 8.40	19.08 ± 9.25	23.87 ± 7.44	29.32 ± 9.18	14.15	0.003

The Kruskal–Wallis test showed significant differences between the four groups.

### 3.3 PAVFs treatment effect and follow-up

**Figure 3:**
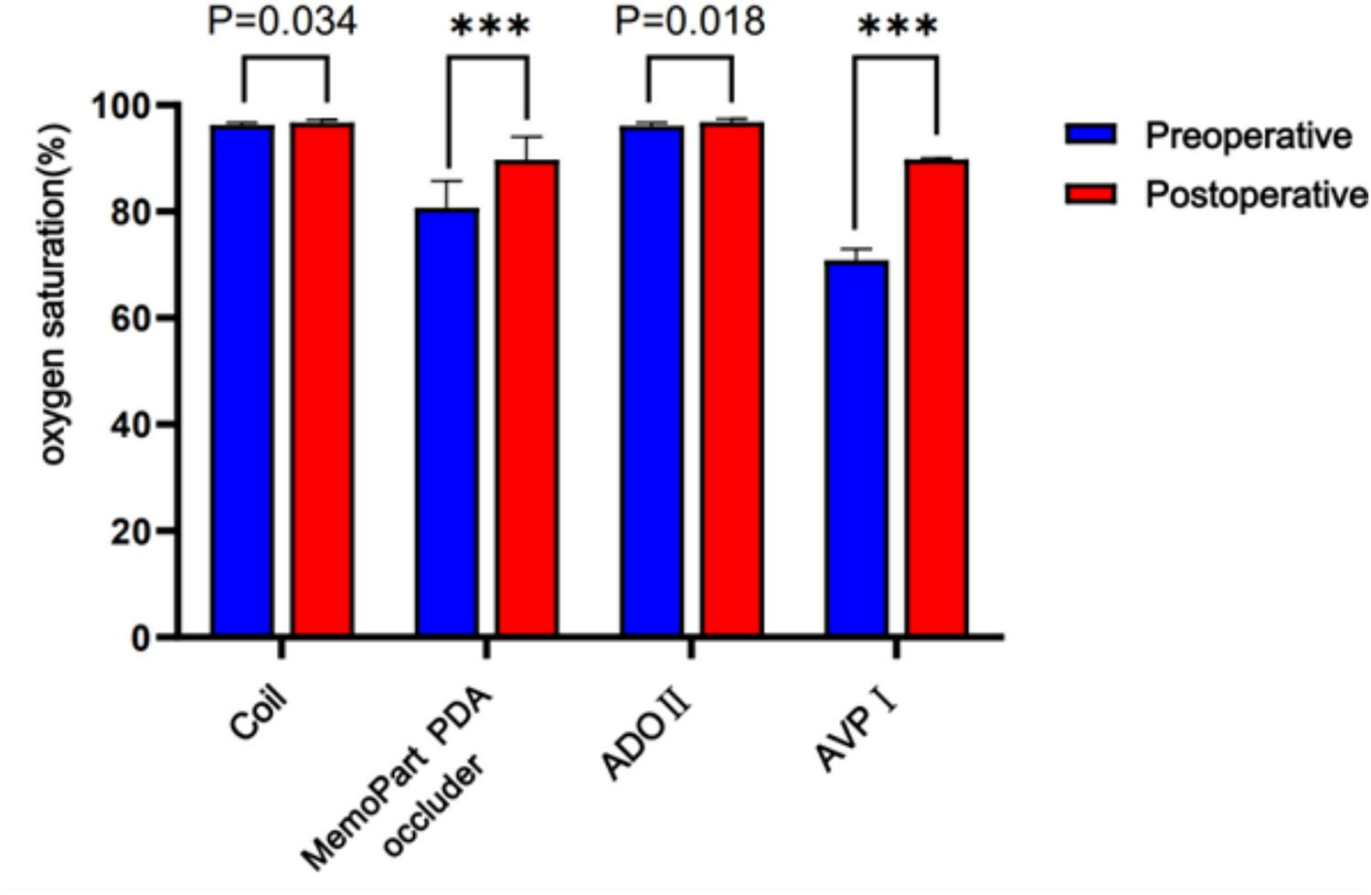
Changes of arterial oxygen saturation before and after operation with different embolization materials; ***P < 0.001.

The preoperative median oxygen saturation was 84.10% (74.00%, 95.00%). After embolization, the blood oxygen saturation level increased to 92.00% (89.00%, 96.55%), with a statistically significant difference (*P* < 0.001). All four groups exhibited elevated SaO_2_ postoperatively (Figure 3). Minor complications occurred in 3 patients (10.71%) immediately after the procedure, all in the MemoPart PDA occluder group: 2 patients (7.14%) of postoperative fever, and 1 patient (3.57%) of ipsilateral pleural effusion at the surgical site. There was no significant difference in complications between the four groups (*P* = 0.848). The mean clinical follow-up duration was 19.46 ± 26.04 months (range, 6–162 months). At the 6-month postoperative follow-up, CTA revealed reperfusion in 1 case (3.45%) from the MemoPart PDA occluder group. No reperfusion was observed in the other three groups (*P* = 0.463) (Table 3). No embolization device migration, detachment, or *in-situ* thrombosis was identified. No other procedure-related complications or deaths occurred during follow-up. Clinical symptoms improved significantly in all patients after transcatheter embolization. The detailed improvement in symptoms is summarized in Supplementary Table S2. All patients achieved varying degrees of relief, including resolution or reduction of dyspnea, cyanosis, hemoptysis, and migraine. Notably, no recurrent stroke was observed in patients with a preoperative history of stroke during follow-up.

**Table 3:** Reperfusion of 41 cases of PAVFs.

Reperfusion status	Coil	MemoPart PDA occluder	ADO Ⅱ	AVP Ⅰ	Total
Reperfusion	0	0	1	0	1
No reperfusion	6	22	6	6	40
Total	6	22	7	6	41

Fisher's exact test showed that there was no significant difference in reperfusion rate among the four groups (*P* = 0.463).

The remaining 28 untreated lesions were categorized as follows: 18 lesions were microscopic PAVFs and no significant shunt; 10 lesions were diffuse micro-shunt lesions unsuitable for one-stage embolization due to extensive distribution and unclear feeding arteries. All untreated lesions were followed up annually with chest CTA, and no significant enlargement or aggravated shunting was observed during follow-up.

## 4 Discussion

Patients with PAVFs are susceptible to a variety of neurological, cardiovascular, respiratory, and hematologic complications. Therefore, prompt diagnosis and intervention are crucial for preventing complications and improving prognosis (11). The primary treatment strategies for PAVFs include surgical resection and interventional embolization. Prior to 1977, surgical interventions were the exclusive treatment option, involving procedures such as surgical ligation of fistulous vessels, excision of fistulous sacs, pulmonary wedge resection, lobectomy, segmentectomy, and pneumonectomy (12). In 1977, the first successful transcatheter embolization for PAVFs was conducted. With the advancement of interventional techniques and medical devices, interventional therapy has emerged as the preferred method for managing PAVFs. For patients with multiple PAVFs, factors such as safety, feasibility, palliative embolization, and patient preference are considered, and embolization is typically performed gradually and in stages (13). In this study, priority was given to addressing fistulas that significantly impacted patient symptoms or were particularly amenable to interventional treatment. The remaining fistulas were evaluated for treatment based on the outcomes of regular follow-up examinations. A cohort of 28 patients was included, and a total of 69 pulmonary arteriovenous fistulas were identified via pulmonary angiography. Of these, 41 fistulas underwent interventional treatment, with all successfully receiving occluders. Five cases exhibited minimal residual shunts, deemed negligible. Consequently, the technical success rate in this study was 100%, with an immediate complete occlusion rate of 87.8%. In comparison, Yu et al. (14) reported higher immediate occlusion rate (98.3% vs. our 87.8%) but significantly higher persistence rate (13.2% vs. our 2.44%). The devices utilized, including coils, Amplatzer series occluders/vascular plugs, and PDA occluders, were largely consistent with those employed in this study. Current studies suggest that the observation period for assessing the occlusion effect may need to be extended during the procedure. Additionally, the selection of varied types of occlusion devices should be tailored to the vessel diameter, and close follow-up is recommended to enhance occlusion efficacy and reduce the rate of recanalization. Historically, patients with feeding arteries less than 3 mm in diameter were considered asymptomatic and thus did not require treatment. Only those with feeding arteries exceeding 3 mm were eligible for intervention, as according to the 3-mm guideline. However, recent studies have indicated that patients with feeding arteries smaller than 3 mm can experience clinical symptoms related to paradoxical embolism. Importantly, the occurrence of paradoxical embolism does no correlation with the diameter of the feeding artery (15). The 2009 International Guidelines for Diagnosis and Management of HHT recommend that PAVFs with feeding arteries less than 3 mm should also be considered for treatment to prevent paradoxical embolism (16). The 2020 CIRSE practice standards (8) suggest embolization for all treatable PAVF. In this study, three PAVFs cases with feeding vessel diameters less than 3 mm underwent intervention, all of which achieved complete occlusion following coil placement.

The selection of embolization material is determined by factors such as the diameter of the target vessel, anatomical morphology, the operator's experience, and the availability of devices at various stages of research (8). Historically, detachable balloons served as the primary material for vascular embolization. However, their use was associated with significant risks, including vascular reperfusion and non-target embolization, due to the large delivery systems, poor controllability, and high retraction rates. Consequently, these risks led to their removal from the U.S. market in 2002 (17). Subsequently, coils have become widely adopted for embolization procedures. These coils are characterized by a diverse range of specifications and ease of operation. Nevertheless, they present certain drawbacks, such as distal displacement, a high rate of continuous shunting, and a significant likelihood of reperfusion (18). In clinical practice, micro PAVFs with small vessel diameters and tortuous paths are are typically managed using coil embolization. Furthermore, its three-dimensional structure enhances the stability of embolization in small vessels, and polypropylene fibers accelerate thrombus formation. In addition to other commonly utilized embolization materials, PDA occluders and vascular plugs, such as the Amplatzer Vascular Plug (AVP) and Micro Vascular Plug (MVP), have been shown to significantly reduce reperfusion rates (19, 20). A study by Letourneau-Guillon et al. (21) reported a mid-term recanalization rate of only 7% for the AVP. The AVP's ability to be repositioned allows for precise distal embolization, minimizing the risk of non-target embolization of adjacent normal vessels. In our study, all six PAVFs treated with AVP I exhibited no recanalization during follow-up. The MVP, compatible with microcatheters, facilitates the treatment of PAVFs with feeding arteries less than 3 mm in diameter, and even as small as 1.5 mm. Moreover, for PAVFs characterized by large feeding artery diameters or complex angioarchitecture, single embolic materials often fail to achieve complete and durable occlusion. As a result, combined embolization strategies are increasingly being adopted (18). This study encompasses a period of 21 years (2003–2024), during which significant advances were achieved in interventional techniques and embolic materials. Initially, coils were predominantly utilized; however, the advent of novel occluders, such as PDA occluders and vascular plugs, enabled the management of more complex and larger-diameter fistulas with enhanced safety and efficacy. The mean diameters of feeding arteries in this study were 3.17 ± 0.7 mm (range: 2.48–4.09 mm) in the coil group, 11.86 ± 3.31 mm (range: 6.42–16.4 mm) in the MemoPart PDA occluder group, 5.98 ± 2.54 mm (range: 3.64–10.5 mm) in the ADO II group, and 8.72 ± 3.78 mm (range: 4.2–12.7 mm) in the AVP I group, with the differences being statistically significant (*P* < 0.001). Although this study is a retrospective analysis and the selection of devices was contingent upon their availability at the time and the anatomical characteristics of the lesions, the overall surgical success rate and safety remained consistently high throughout the study period. This suggests that the fundamental principles of TCE therapy are robust, and that technological advancements, by providing a broader array of devices, further optimize individualized treatment strategies, potentially improving long-term outcomes. Based on the data from this study and existing literature, the following recommendations are proposed for selecting embolization devices for PAVF treatment (8, 22). The coil is appropriate for feeding arteries with diameters less than 4 mm, and it is advised that the device's diameter exceed the vessel diameter by 1–2 mm. The MemoPart PDA occluder is suitable for feeding arteries with diameters ranging from 6 to 16 mm or greater, with a recommended device diameter that is approximately 40% larger than the vessel diameter. The ADO II is suitable for feeding arteries measuring between 4 and 10 mm. And its disc diameter should exceed the vessel diameter by 4–6 mm, while the waist diameter should closely match the vessel diameter. The AVP I is appropriate for feeding arteries with diameters between 4 and 12 mm, and its diameter should be approximately 40% larger than the vessel diameter.

Transcatheter embolization for PAVFs demonstrates a low complication rate. The most common postoperative complications are fever and pleurisy, occurring within 1–2 days following the procedure, with an incidence ranges from 15% to 30%. These symptoms typically persist for 4–6 days and can be effectively managed with oral nonsteroidal anti-inflammatory drugs (NSAIDs) (8). In our case series, minor complications were observed immediately post-procedure in 3 patients (10.71%). No additional procedure-related complications were noted during follow-up, and there were no procedure-related mortalities. This complication incidence aligns with findings from previous studies (23). Recurrence of PAVFs post-embolization may be associated with recanalization or enlargement of previously untreated micro-PAVFs (24). In this study, 2 out of 41 PAVFs (4.88%) required further embolization due to the growth of initially untreatable micro-PAVFs. Recanalization remains the most common cause of recurrence and necessitates retreatment. The lower recanalization rate observed in our study (2.44%) underscores the importance of individualized device selection, corroborating the findings of Srinivas and colleagues (25).

Management of special types of PAVFs: 1. PAVFs with HHT: PAVFs linked to HHT are characterized by multifocality and high recurrence rates. Research indicates that genetic testing, such as for ENG, ALK1 mutations, can guide therapeutic strategies. In patients with ALK1 mutations, the use of anti-VEGF agents, such as, bevacizumab, thalidomide, may delay lesion progression. Additionally, tyrosine kinase inhibitors (TKIs) potentially suppress VEGF signaling, thereby preventing capillary dilation and arteriovenous malformation formation (26). 2. Pediatric and pregnant patients: In pediatric populations, PAVFs are predominantly hereditary. Treatment is recommended for large PAVFs, defined by an afferent artery diameter of ≥3 mm, and for those associated with reduced oxygen saturations in children (27). During pregnancy, there is an increased risk of PAVF rupture. Therefore, pre-gestational embolization is strongly recommended. If clinically necessary, emergency embolization during the second trimester may be considered.

### 4.1 Limitations

This study is a single-center retrospective analysis characterized by a limited sample size, which introduces potential risk of selection biases and information biases. Conducted over a 21-year period, the study may be affected by variations in embolization devices and techniques, as well as significant variation in follow-up duration, potentially compromising data homogeneity. Certain diffuse PAVFs received only palliative embolization without achieving complete occlusion, which could influence the outcomes of long-term follow-up. Moreover, cardiopulmonary exercise test (CPET) and 6-minute walk test (6MWT) were not routinely performed, leading to absent adequate functional assessment. To substantiate the long-term efficacy of various embolic materials, future research should focus on multicenter prospective studies.

## 5 Conclusion

In summary, the strategic application of various occluders can significantly improve the success rate of achieving effective occlusion. Transcatheter embolization for PAVFs demonstrates high safety profile and efficacy, characterized by superior procedural success rates and enduring long-term outcomes. This approach effectively ameliorates symptoms of right-to-left shunting and mitigates or prevents neurological complications associated with PAVFs.

## Data Availability

The datasets presented in this article are not readily available because data supporting the ﬁndings of this study are encrypted and stored in military cardiovascular intervention diagnosis and treatment management information network of China. Requests to access the data should be directed to the corresponding author.
