# Supplementary material for: Safety and efficacy of transcatheter embolization for pulmonary arteriovenous fistula: a 21-year retrospective study
Source: Front Cardiovasc Med. 2026 May 29;13:1732994. doi: 10.3389/fcvm.2026.1732994 (PMC13260122; doi:10.3389/fcvm.2026.1732994)
Supplement: Supplementary file 1 [file Table1.docx]

**Table S1** **Preoperative hemodynamic parameters in the overall cohort and four subgroups**

|  | **Overall** | **Coil** | **MemoPart PDA occluder** | **ADO Ⅱ** | **AVP Ⅰ** | **H-value** | **P-value** |
| --- | --- | --- | --- | --- | --- | --- | --- |
| **mPAP (mmHg)** | 17.71±4.12 | 18.72±3.15 | 17.99±4.83 | 17.71±3.84 | 15.67±2.00 | 1.767 | 0.622 |
| **mRAP (mmHg)** | 4.68±2.22 | 5.33±1.97 | 4.62±2.64 | 3.86±0.90 | 5.22±1.87 | 4.022 | 0.259 |
