# Supplementary material for: Safety and efficacy of transcatheter embolization for pulmonary arteriovenous fistula: a 21-year retrospective study
Source: Front Cardiovasc Med. 2026 May 29;13:1732994. doi: 10.3389/fcvm.2026.1732994 (PMC13260122; doi:10.3389/fcvm.2026.1732994)
Supplement: Supplementary file 2 [file Table2.docx]

Table S2 Clinical symptom improvement following transcatheter embolization

| **Symptom** | Complete relief, n (%) | Partial relief, n (%) |
| --- | --- | --- |
| Dyspnea (n=13) | 1 (7.69) | 12 (92.31) |
| Cyanosis (n=13) | 1 (7.69) | 12 (92.31) |
| Hemoptysis (n=3) | 1 (33.33) | 2 (66.67) |
| Migraine (n=7) | 0 (0) | 7 (100) |
| Stroke (n=5) | 5 (100)^a^ | 0 (0) |

^a^ Complete relief of stroke was defined as no recurrence of ischemic stroke during follow-up.
